# Whole Brain Radiation Therapy Plus Focal Radiation Boost May Generate Better Survival Benefit for Brain Metastases From Non-small Cell Lung Cancer

**DOI:** 10.3389/fonc.2020.576700

**Published:** 2020-10-20

**Authors:** Meng Ni, Wenju Liu, Aijun Jiang, Yong Wang, Yanxing Sheng, Haiyan Zeng, Ning Liu, Li Li, Yiqiang Qi, Yu Wang, Jinming Yu, Shuanghu Yuan

**Affiliations:** ^1^ Department of Radiation Oncology, Qingdao University Medical College Affiliated Yantai Yuhuangding Hospital, Yantai, China; ^2^ Department of Radiation Oncology, Shandong Cancer Hospital and Institute, Shandong First Medical University and Shandong Academy of Medical Sciences, Jinan, China; ^3^ Department of Radiation Oncology, The Affiliated Hospital of Xuzhou Medical University, Xuzhou, China; ^4^ Department of Radiation Oncology, Liaocheng People’s Hospital, Liaocheng, China; ^5^ Department of Radiation Oncology, ZiBo Central Hospital, Zibo, China; ^6^ Department of Radiation Oncology, Shandong Cancer Hospital and Institute-Shandong Cancer Hospital Affiliated to Shandong University, Jinan, China

**Keywords:** brain metastases, non-small cell lung cancer, whole brain radiation therapy, whole brain radiation therapy plus focal boost, stereotactic radiosurgery

## Abstract

**Background:**

Owing to improved systemic therapies, the survival of patients with non-small cell lung cancer (NSCLC) was prolonged, and the risk of brain metastases was consequently increased. This study aims to compare different radiotherapy for brain metastases in patients with NSCLC.

**Materials and methods:**

The patients with NSCLC who were treated with whole brain radiation therapy (WBRT) or stereotactic radiosurgery (SRS) for brain metastases at three medical centers between January 2012 and December 2017 were retrospectively analyzed.

**Results:**

Of the 684 eligible patients, 217 received WBRT plus focal radiation boost (WBRT+boost), 324 received WBRT, and 143 received SRS. Patients with WBRT+boost lived longer than those with WBRT (median overall survival (OS), 22.2 vs 13.7 months, *P* < 0.001) or SRS (22.2 vs 17.3 months, *P* = 0.011). In subgroup analyses, the survival advantage of WBRT+boost was more obvious among patients with 1 to 3 brain metastases or who received targeted therapy than did SRS. From pair-wise comparisons of intracranial progression-free survival (iPFS), WBRT+boost was also superior to WBRT (12.9 vs 10.6 months, *P* = 0.028) and SRS (12.9 vs 9.1 months, *P* = 0.001).

**Conclusions:**

Patients who were treated with WBRT+boost experienced significantly longer OS and iPFS than those with WBRT or SRS alone. WBRT+boost should be a preferred strategy for brain metastases in NSCLC patients.

## Introduction

Brain metastasis is the most common source of central nervous system tumors, and the majority of brain metastases originate from non-small cell lung cancer (NSCLC) (1, 2). The prognosis of NSCLC patients with brain metastasis is poor. With the best supportive care (dexamethasone), the median overall survival (OS) is only 8.5 weeks (3). In 1970 to 1990s, whole brain radiation therapy (WBRT) became the standard treatment for brain metastasis, which doubled the median OS to 16 weeks (4). However, no studies were conducted to identify whether other altered fractionation schemes could obtain a longer survival benefit or less neurotoxicity than the scheme of 30Gy in 10 fractions (5). Since 1990s, the treatment of brain metastasis evolved localized therapies such as surgery, stereotactic radiosurgery (SRS), three-dimensional conformal radiotherapy (3D-CRT), intensity-modulated radiation therapy (IMRT), or combinations of the above treatments, together with systemic therapies, such as chemotherapy, molecular targeted therapy, and immunotherapy (6, 7). With these treatments, median OS has been prolonged to more than 10 months (8, 9). For patients with drug-sensitive gene mutations, tyrosine kinase inhibitor (TKI) therapy could even prolong the median OS to 46 months (10). Owing to various of effective systemic therapies, the risk of brain metastases was also increased because of the prolonged survival. Currently, whether WBRT should be administered or not is still of considerable controversy. In order to decrease the neurotoxicity, some investigators suggested that SRS should/might be a standard treatment for limited brain metastases (up to 4 brain metastases), and even for multiple brain metastases (5–10 brain metastases) (8, 11, 12). In order to improve the intracranial control rate, some investigators proposed the use of WBRT plus focal radiation boost (13, 14). Therefore, it is important to identify a better strategy for brain metastasis in patients with NSCLC.

## Materials and Methods

### Data Collection

**Figure 1:**
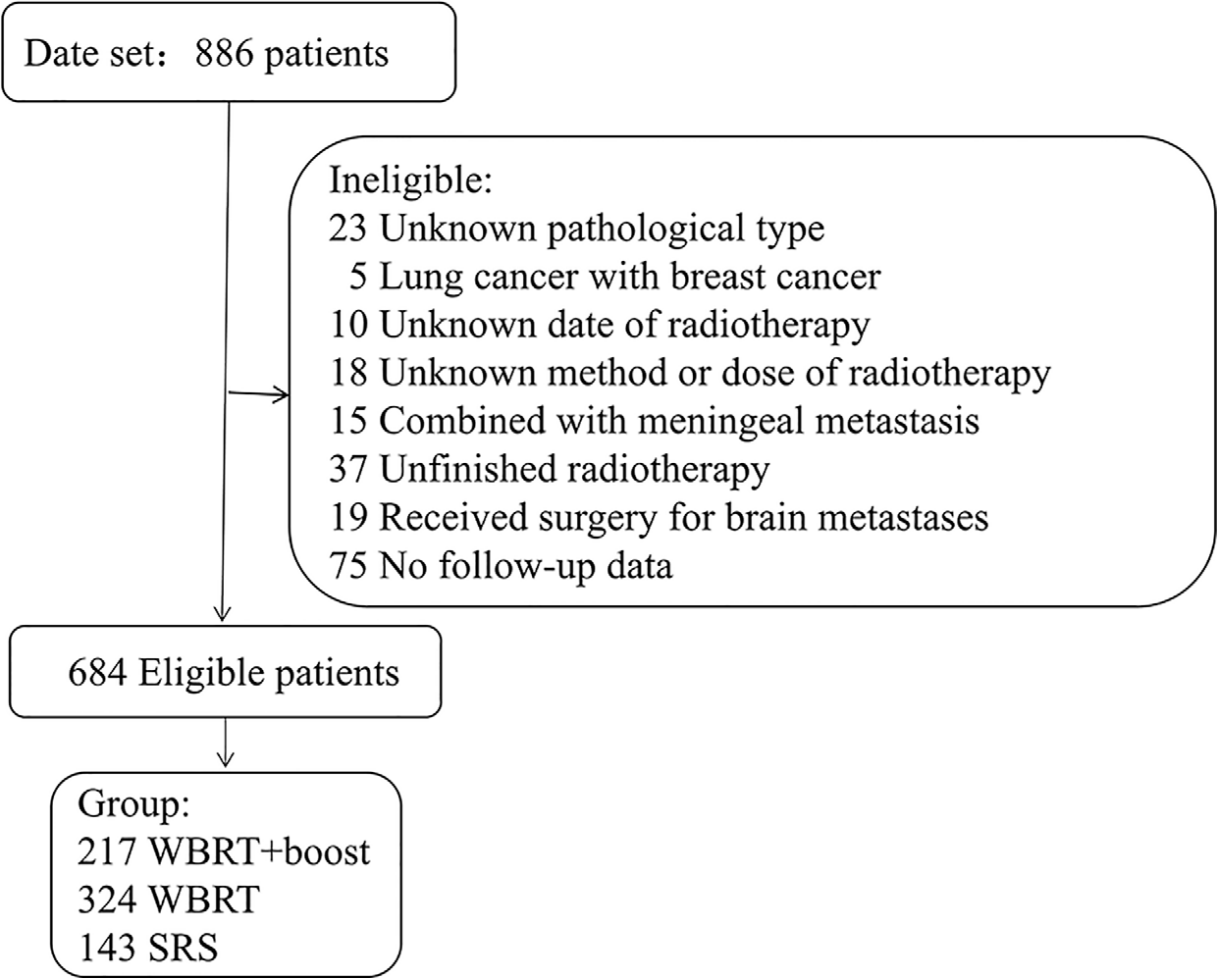
Patients screened and determined to be, eligible or ineligible for inclusion in the study. Abbreviations: WBRT+boost, WBRT plus focal radiation boost.

This was a multi-center retrospective study. The NSCLC patients who treated with WBRT or SRS for brain metastases in three medical institutions between January 2012 and December 2017 were reviewed. The eligible criteria included pathologically identified NSCLC and imaging (MRI/CT) identified brain metastases. All patients were treated with WBRT or SRS for brain metastases. Cranial MRI/CT surveillance was performed every 2 to 3 months after brain radiotherapy, then every 5 to 6 months after 3 years. Patients with incomplete medical records or those who did not complete the prescribed treatment were excluded (**Figure 1**). According to the accepted brain radiotherapy, the remaining 684 patients were divided into three groups: the WBRT plus focal radiation boost group (WBRT+boost, n = 217), WBRT group (n = 324), and SRS group (n = 143). The general characteristics were recorded, including gender, age, Karnofsky Performance Status (KPS), diagnosis-specific Graded Prognostic Assessment (DS-GPA), gene mutations, number of brain metastases, maximum size of brain metastasis, systemic treatment, type of radiotherapy, and extracranial metastases.

### Study Treatment

WBRT was performed using 3D-CRT or IMRT techniques, and for 19 patients, WBRT was administered under guidance by 2D-CRT. In WBRT, the clinical target volume (CTV) was contoured as the region of the whole brain, and expansion of the CTV by 3 mm was used as the planning tumor volume (PTV). The radiation dose was 25 to 45 Gy in 10 to 20 fractions, 2 to 3 Gy per fraction. The additional radiation boost following WBRT was delivered by linear accelerators, with the total dose of 10 to 30 Gy in 5 to 15 fractions. The gross tumor volume (GTV) was defined as the range of contrast-enhanced tumor, and expansion of the GTV by 2 to 3 mm was used as the PTV. SRS was performed using gamma knife according to the size of the brain metastasis and the location of the tumor in the functional area, with a 40% to 60% isodose line. The prescribed radiation dose of SRS was 13.5 to 21.0 Gy in 1 to 2 fractions, 8.5 to 16.0 Gy per fraction.

### Study End Points and Statistical Analysis

The primary end point was OS, and the secondary end point was iPFS (the progression of intracranial metastases or death). Disease progression was assessed according to the Response Evaluation Criteria in Solid Tumors version 1.1 criteria (15). All end points were defined as the time from the start of brain radiotherapy to the respective events, which were censored at the final follow-up (data cutoff was December 31, 2018) if no respective events were observed. The general characteristics of patients were calculated by ANOVA or Chi-square test. The OS and iPFS were evaluated by Kaplan–Meier method, and groups were compared using the Breslow test. For pairwise comparisons of OS, multiplicity adjustment was implemented by the sequentially rejective Bonferroni method (16). Planned subgroup analyses of OS among WBRT+boost and SRS group did not adjust for multiplicity adjustment, so these results should be interpreted as exploratory. Univariate and multivariate analyses based on Cox proportional hazards regression models were performed to assess the impact of prognostic variables (17). A two-sided value of *P* < 0.05 was considered as statistically significant. All analyses were performed using IBM SPSS version 22.0 (IBM Corp).

## Results

### Patient Characteristics

Among the 684 included patients, 447 (65.3%) patients had died, 130 (19.0%) patients remained alive, and 107 (15.6%) patients were lost to follow-up. The mean age was 58 years (SD, 10 years); 399 (58.3%) patients were men. The baseline characteristics of patients are presented in **Table 1**.

**Table 1:** Baseline characteristics of the included NSCLC patients with brain metastases.

	WBRT+boost(n = 217)	WBRT Alone(n = 324)	SRS Alone(n = 143)
Age, mean (SD), y	57 (10)	57 (11)	60 (10)
Sex, No.			
Male	124	200	75
Female	93	124	68
KPS, No.			
≤80	145	223	94
>80	72	101	49
Number of BMs, No.			
1–3	122	77	118
>3	95	247	25
Maximum size of BM, No.			
<20 mm	91	181	77
≧20 mm	109	124	57
Unknown	17	19	9
ECM, No.			
Yes	59	131	42
None	27	45	27
Unknown	131	148	74
DS-GPA scores, No.			
0.0–1.0	41	103	13
1.5–2.0	104	165	68
2.5–3.0	66	53	54
3.5–4.0	6	3	8
Targeted Tx, No.			
Yes	82	100	45
None	135	224	98
Total dose, mean (SD), Gy	50 (6)	36 (8)	17 (1)

WBRT+boost, whole brain radiotherapy plus focal radiation boost; WBRT, whole brain radiation therapy; SRS, stereotactic radiosurgery; KPS, Karnofsky Performance Status; BM, brain metastasis; ECM, extracranial metastases; DS-GPA, diagnosis-specific Graded Prognostic Assessment; Tx, treatment.

### Overall Survival

**Figure 2:**
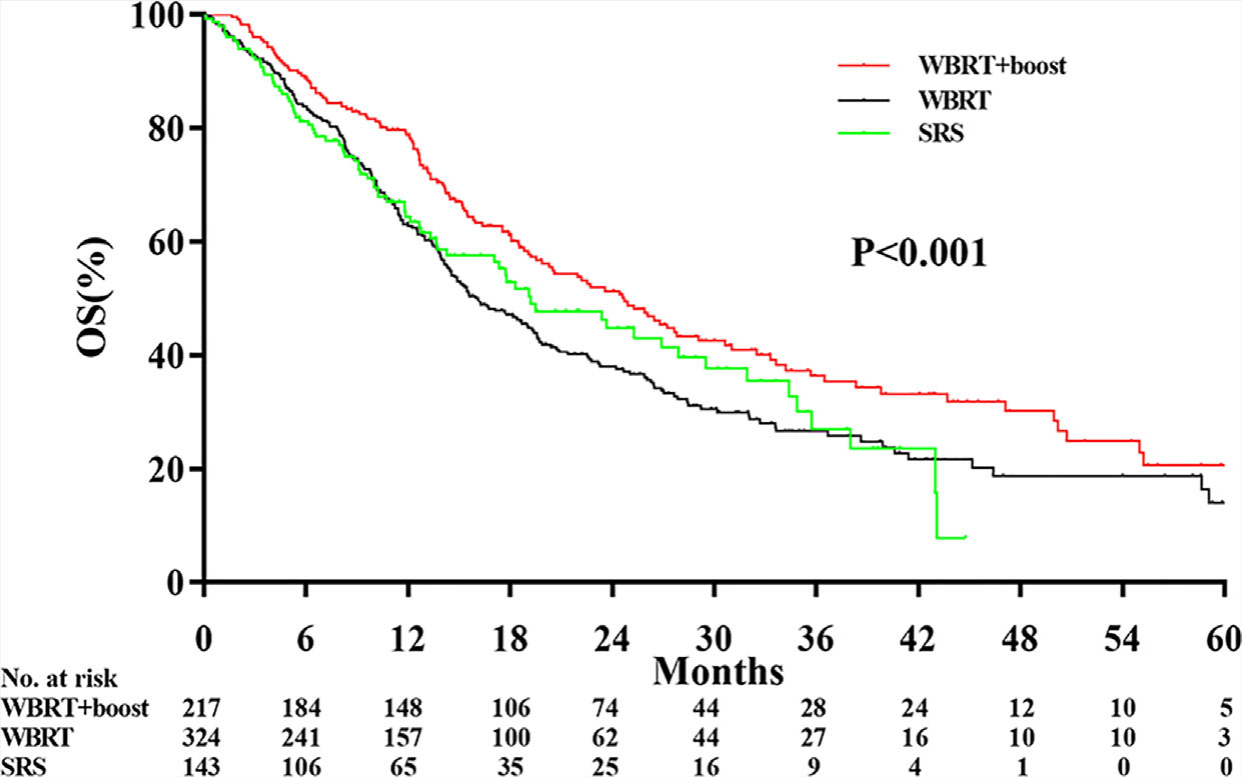
Comparison of overall survival according to the type of adjuvant radiotherapy received. WBRT+boost indicates whole brain radiotherapy plus focal radiation boost; WBRT, whole brain radiotherapy; SRS, stereotactic radiosurgery. There were significant differences in overall survival among the WBRT+boost, WBRT, and SRS groups (22.2 vs 13.7 vs 17.3 months, *P* < 0.001).

The median follow-up time was 13.0 months. Significant differences in OS were observed among the WBRT+boost, WBRT, and SRS groups (22.2 vs 13.7 vs 17.3 months, *P* < 0.001, **Figure 2**). Upon pairwise comparison of the OS rates, WBRT+boost resulted in longer OS than in WBRT (22.2 vs 13.7 months; *P* < 0.001) or SRS (22.2 vs 17.3 months; *P* = 0.011). This finding was also supported by univariate analysis (**Table 2**). Additionally, no significant difference in OS was observed between the WBRT and SRS groups (13.7 vs 17.3 months; *P* = 0.221, **Figure 2**).

**Table 2:** Univariate and multivariate analyses of factors influencing OS of NSCLC patients with brain metastases.

Factors		Median OS, months	Univariate		Multivariate	
			HR (95% CI)	*P-*Value	HR (95% CI)	*P-*Value
Age, years	<60	19.5		<0.001		
	≥60	13.9	1.5 (1.3–1.8)			
Sex	Male	13.9		<0.001		**0.003**
	Female	20.3	0.6 (0.5–0.8)		0.7 (0.6–0.9)	
KPS	≤80	15.5		0.352		
	>80	16.4	0.9 (0.7–1.1)			
Number of BMs	1–3	19.5		0.006		
	>3	14.7	1.3 (1.1–1.6)			
Maximum size of	<20	15.1		0.248		
BM, mm	≥20	16.0	1.0 (0.8–1.2)			
	Unknown	27.8	0.7 (0.5–1.1)			
DS-GPA	0.0–1.0	12.6		<0.001		**0.016**
	1.5–2.0	16.0	0.7 (0.6–0.9)		0.8 (0.6–1.1)	
	2.5–3.0	24.9	0.5 (0.4–0.7)		0.6 (0.4–0.8)	
	3.5–4.0	36.5	0.4 (0.2–0.8)		0.4 (0.2–0.9)	
Targeted Tx	Yes	26.5		<0.001		**<0.001**
	No	14.1	2.0 (1.6–2.5)		2.1 (1.7–2.6)	
Radiotherapy	WBRT+boost	22.2		<0.001		**0.003**
	WBRT	13.7	1.6 (1.3–2.0)		1.4 (1.1–1.8)	
	SRS	17.3	1.4 (1.0–1.8)		1.4 (1.1–1.9)	

OS, overall survival; HR, hazard ratio; CI, confidence interval; KPS, Karnofsky Performance Status; BM, brain metastasis; DS-GPA, diagnosis-specific Graded Prognostic Assessment; Tx, treatment; WBRT, whole brain radiation therapy; SRS, stereotactic radiosurgery; WBRT+boost, whole brain radiotherapy plus focal radiation boost.

### Subgroup Analyses

To further analyze the OS between WBRT+boost and SRS group, subgroup analyses were conducted based on number of brain metastases, and use of systemic treatment. Patients with WBRT+boost experienced a longer OS than SRS in patients with 1 to 3 brain metastases (24.6 vs 14.3 months, *P* = 0.001; **eFigure 1A** in the Supplement), or in patients who received targeted therapy (31.0 vs 17.3 months, *P* = 0.026; **eFigure 2A** in the Supplement). No survival advantage was observed in patients with multiple (>3) brain metastases (19.1 vs 18.3 months, *P* = 0.649; **eFigure 1B** in the Supplement) or in patients who did not receive targeted therapy (19.2 vs 18.3 months, *P* = 0.146; **eFigure 2B** in the **Supplement**).

### Intracranial Progression-free Survival

**Figure 3:**
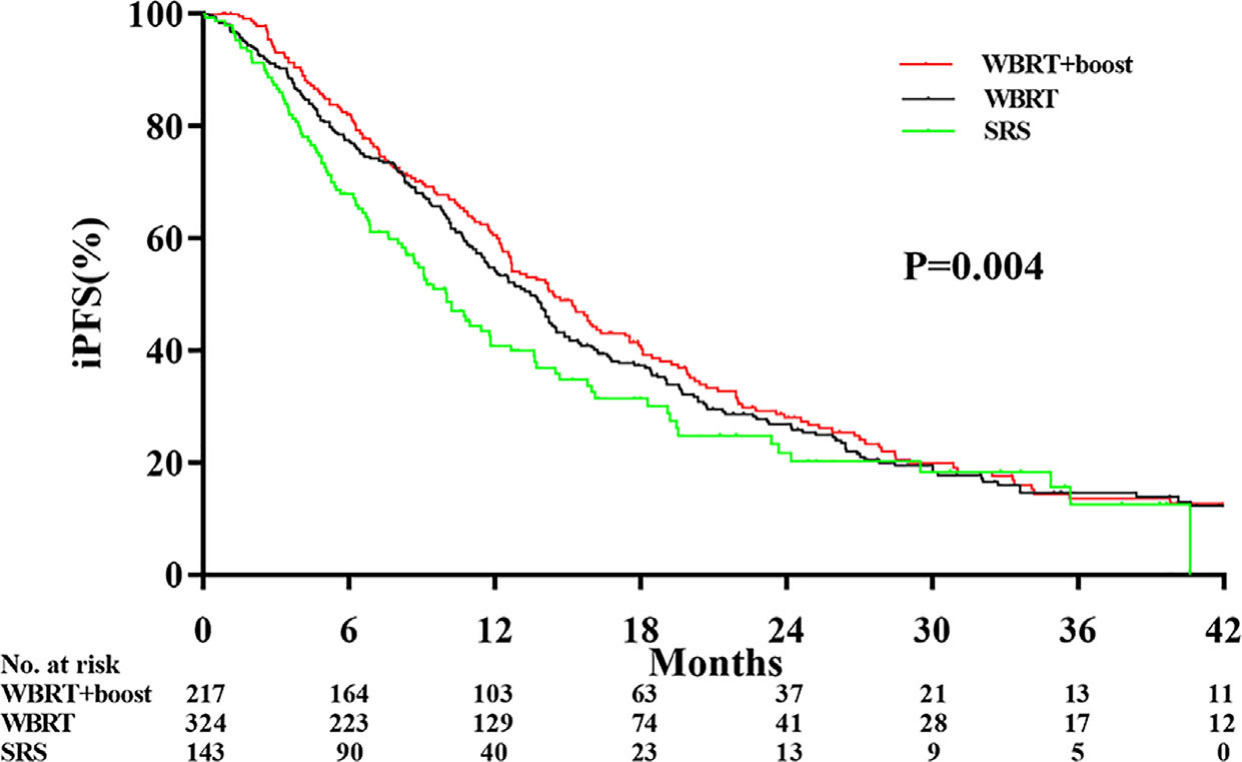
Comparison of intracranial tumor progression-free survival according to the type of adjuvant radiotherapy received. Abbreviations: WBRT+boost indicates whole brain radiotherapy plus focal radiation boost; WBRT, whole brain radiotherapy; SRS, stereotactic radiosurgery. The intracranial tumor progression-free survival time was different among the WBRT+boost, WBRT alone, and SRS alone groups (12.9 vs 10.6 vs 9.1 months, *P* = 0.004).

Significant differences in the iPFS were observed among the WBRT+boost, WBRT, and SRS groups (12.9 vs 10.6 vs 9.1 months, *P* = 0.004; **Figure 3**). From pairwise comparisons of groups, the iPFS of WBRT+boost group was significantly longer than WBRT (12.9 vs 10.6 months, *P* = 0.028) or SRS (12.9 vs 9.1 months, *P* = 0.001). No significant difference in iPFS was observed between the WBRT and SRS groups (10.6 vs 9.1 months, *P* = 0.108; **Figure 3**).

### Univariate and Multivariate Analyses of Survival

Statistically significant covariates in univariate analysis were further analyzed by multivariate Cox proportional hazards analyses. In multivariate analyses for OS, female sex, high DS-GPA score (1.5–4), targeted therapy, and WBRT+boost were significant protective factors. The mortality rates in patients who were treated with WBRT and SRS alone were about 1.4-fold greater than those with WBRT+boost. KPS scores, the size and number of brain metastases were not significantly associated with OS (**Table 2**). Additionally, sex, DS-GPA score, systemic therapy, and the type of radiotherapy were also significant prognostic factors for iPFS (**Table 3**).

**Table 3:** Univariate and multivariate analyses of factors influencing iPFS in NSCLC patients with brain metastases.

Factors		Median iPFS, months	Univariate		Multivariate	
			HR (95% CI)	*P-*Value	HR (95% CI)	*P-*Value
Age	<60	11.6		0.027		
	≥60	10.2	1.2 (1.0–1.4)			
Sex	Male	9.5		<0.001		**0.004**
	Female	13.4	0.7 (0.6–0.9)		0.8 (0.6–0.9)	
KPS	≤80	11.1		0.783		
	>80	10.6	1.0 (0.9–1.2)			
Number of BMs	1–3	11.1		0.294		
	>3	10.8	1.1 (0.9–1.3)			
Maximum size of	<20	10.5		0.495		
BM, mm	≥20	11.2	1.0 (0.9–1.2)			
	Unknown	14.2	0.8 (0.6–1.2)			
DS-GPA	0.0–1.0	9.0		0.002		**0.010**
	1.5–2.0	11.2	0.7 (0.6–0.9)		0.7 (0.5–0.9)	
	2.5–3.0	12.7	0.6 (0.5–0.8)		0.5 (0.4–0.8)	
	3.5–4.0	10.0	0.8 (0.4–1.3)		0.6 (0.3–1.1)	
Targeted Tx	Yes	14.0		<0.001		**<0.001**
	No	10.0	1.5 (1.2–1.8)		1.4 (1.2–1.7)	
Radiotherapy	WBRT+boost	12.9		0.047		**0.049**
	WBRT	10.6	1.2 (1.0–1.4)		1.1 (0.9–1.3)	
	SRS	9.1	1.3 (1.1–1.7)		1.4 (1.1–1.8)	

iPFS, intracranial progression-free survival; HR, hazard ratio; CI, confidence interval; BM, brain metastasis; DS-GPA, diagnosis-specific Graded Prognostic Assessment; Tx, treatment; WBRT, whole brain radiation therapy; SRS, stereotactic radiosurgery; WBRT+boost, whole brain radiotherapy plus focal radiation boost.

## Discussion

Our data showed that WBRT+boost resulted in significantly longer iPFS and OS than did WBRT or SRS, in agreement with other reported series (9, 18). For iPFS, Rades et al. found that WBRT plus stereotactic boost achieved better iPFS at 1-year than WBRT (71% vs 48%, *P* = 0.005) (18). Additionally, Brown et al. reported that patients who were treated with WBRT plus SRS was associated with significantly higher 1-year intracranial control rate than SRS alone (90.1% vs 72.8%, *P* = 0.003) (9). As for OS, some studies observed a survival advantage in WBRT+boost group over WBRT group (13, 19). In the RTOG 9508 randomized clinical trial, Andrews et al. found that compared to WBRT alone, WBRT plus SRS increased the OS in patients with single brain metastasis (6.5 vs 4.9 months, *P* = 0.039) (13). Sun et al. also found that WBRT plus radiation boost increased OS than WBRT alone in SCLC patients (13.4 vs 8.5 months, *P* = 0.004) (19). Of note, none of the above studies only included NSCLC patients.

The most important finding in this study was that WBRT+boost resulted in significantly longer iPFS and OS than SRS. To further analyze the survival difference between WBRT+boost and SRS group, several subgroup analyses were performed. Compared to SRS in patients with either 1 to 3 brain metastases, WBRT+boost improved survival (25.3 vs 17.8 months, *P* < 0.001). Similarly, compared to patients who received targeted therapy, WBRT+boost also improved survival (32.5 vs 23.7 months, *P* = 0.033). These results challenged the current guidelines for brain metastases. For example, National Comprehensive Cancer Network (NCCN) guidelines version 7·2019 recommend SRS alone for patients with limited brain metastases. WBRT or additional focal boost was totally abandoned in the treatment for these patients. This strong recommendation was originated from Brown’s famous randomized clinical trial published on JAMA 2016. Although they reported that the SRS plus WBRT lead to much lower cumulative incidence of intracranial tumor progression than SRS alone (6.3% vs 24.7% at 3 months, 11.6% vs 35.3% at 6 months, and 15.0% vs 49.5% at 12 months; *P* < 0.001), there was no significant difference in OS (7.4 vs 10.4 months, *P* = 0.92). Thus, they concluded that SRS alone were a preferred strategy in patients with limited brain metastases (9). The conclusion was then widely accepted by oncologists because it originated from the multicenter randomized clinical trial, and was therefore cited in NCCN guidelines. WBRT has been abandoned because of the side effects of cognitive impairment (20). Notably, the median OS in our study were much better than those reported by Brown et al. (9). Such improvement in OS could possibly be attributed to the use of multidisciplinary comprehensive treatments, particularly the targeted therapy (8, 21). According to the published articles included in **Table 4**, the median OS in NSCLC patients with brain metastases has been prolonged by administration of targeted therapy (8–10, 13, 21). Other researches have showed that prolonged survival can also result in higher incidence of intracranial metastasis recurrence, which was always associated with a neurologic deficit (22–24). With the improved extracranial control by effective targeted therapy or other systematic therapy, the high intracranial tumor control rate following WBRT+boost may translate into OS benefit. Therefore, this study suggested that WBRT+boost might be a preferred strategy for brain metastases in NSCLC patients, especially in patients with 1 to 3 brain metastases in the era of effective systematic therapy.

**Table 4:** Literature review of adjuvant treatments with radiotherapy or TKI for brain metastases.

First author, year of publication	Study period	No. of BMs	RT and systemic treatment	Median iPFS, months	Median OS, months
Andrews, 2004 (13)	1996–2001	1–3	WBRT vs SRS+WBRT	–	4.9 vs 6.5
Iuchi, 2013 (8)	2007–2012	1–4	Gefitinib only	14.5	21.9
Brown, 2016 (9)	2002–2013	1–3	SRS vs SRS+WBRT	–	10.4 vs 7.4
Yang, 2017 (21)	2012–2015	>3	WBRT vs Icotinib	10.0 vs 4.8	20.5 vs 18.0
William, 2017 (10)	2008–2014	Unknown	SRS+TKI vs WBRT+TKI vs TKI+WBRT/SRS	23 vs 24 vs 17	46 vs 30 vs 25

BM, brain metastasis; RT, radiation therapy; OS, overall survival; iPFS, intracranial progression-free survival; WBRT, whole brain radiation therapy; SRS, stereotactic radiosurgery; TKI, tyrosine kinase inhibitor.

Of course, we did not ignore the cognitive decline caused by WBRT, too. NRG Oncology CC001 trial showed that in the absence of differences in OS and intracranial PFS, hippocampus sparing during WBRT plus memantine significantly reduced the risk of cognitive failure than did WBRT plus memantine (HR, 0.74; 95% CI, 0.58–0.95; *P* = 0.02) (25). RTOG 0933 trail also showed that hippocampus sparing during WBRT was associated with preservation of neurocognitive function (26). Hippocampus sparing during WBRT plus focal boost may be associated with longer OS and lower neurotoxicity, of course, which needs to be verified by prospective randomized clinical trial.

Our present study also has several limitations. Firstly, the inherent characteristics of retrospective research and patient heterogeneity may have led to bias in the results. Second, the average prescription dose in this study was lower than RTOG prescription isodose (27), which may bias the results. Thirdly, this retrospective study did not evaluate radiation-related neurotoxicity.

## Conclusions

Our data showed that for NSCLC patients who experienced brain metastases, WBRT+boost is associated with better OS and iPFS than WBRT or SRS alone. The additional focal radiation boost is not accompanied by increased neurotoxicity, the adjuvant WBRT is always associated with better intracranial local control. Therefore, WBRT+boost should be considered for NSCLC patients with brain metastases.

## Data Availability Statement

The raw data supporting the conclusions of this article will be made available by the authors, without undue reservation.

## Ethics Statement

The studies involving human participants were reviewed and approved by The Ethics Committee of Shandong Cancer Hospital, The Ethics Committee of Liaocheng People's Hospital, The Ethics Committee of The Affiliated Hospital of Xuzhou Medical University . The patients/participants provided their written informed consent to participate in this study.

## Author Contributions

SY, JY, and MN contributed conception and design of the study. MN, WL, AJ, YoW, YS, HZ, NL, LL, YQ, and YuW organized the database. MN, WL, AJ, YoW, and YQ performed the statistical analysis. MN, WL, AJ, and LL wrote the first draft of the manuscript. YoW, YS, HZ, and NL wrote sections of the manuscript. All authors contributed to the article and approved the submitted version.

## Funding

This work was supported by Natural Science Foundation of China (NSFC81872475, NSFC81372413), Shandong Key Research and Development Plan (2017CXGC1209, 2017GSF18164) and the Outstanding Youth Natural Science Foundation of Shandong Province (JQ201423), Jinan Clinical Medicine Science and Technology Innovation Plan (201704095), National Key Research and Development Program of China (2016YFC0904700).

## Conflict of Interest

The authors declare that the research was conducted in the absence of any commercial or financial relationships that could be construed as a potential conflict of interest.

## Supplementary Material

Supplementary Figure 1 Overall survival impact of number of brain metastases. Abbreviations: WBRT+boost indicates whole brain radiotherapy plus focal radiation boost; SRS, stereotactic radiosurgery. The plots showed that compared to SRS alone, a significant survival benefit of WBRT+boost in patients with 1 to 3 brain metastases (A) (24.6 vs 14.3 months, *P* = 0.001).

Supplementary Figure 2 Comparison of overall survival among subgroups based on the administration of systemic treatment. Abbreviations: WBRT+boost indicates whole brain radiotherapy plus focal radiation boost; SRS, stereotactic radiosurgery. The plots showed a significant difference in OS was observed in patients who received targeted therapy (A) in the comparison of groups (31.0 vs 17.3 months, *P* = 0.026).
