# Supplementary figures and images for: Whole Brain Radiation Therapy Plus Focal Radiation Boost May Generate Better Survival Benefit for Brain Metastases From Non-small Cell Lung Cancer
Source: Front Oncol. 2020 Oct 20;10:576700. doi: 10.3389/fonc.2020.576700 (PMC7606935; doi:10.3389/fonc.2020.576700)

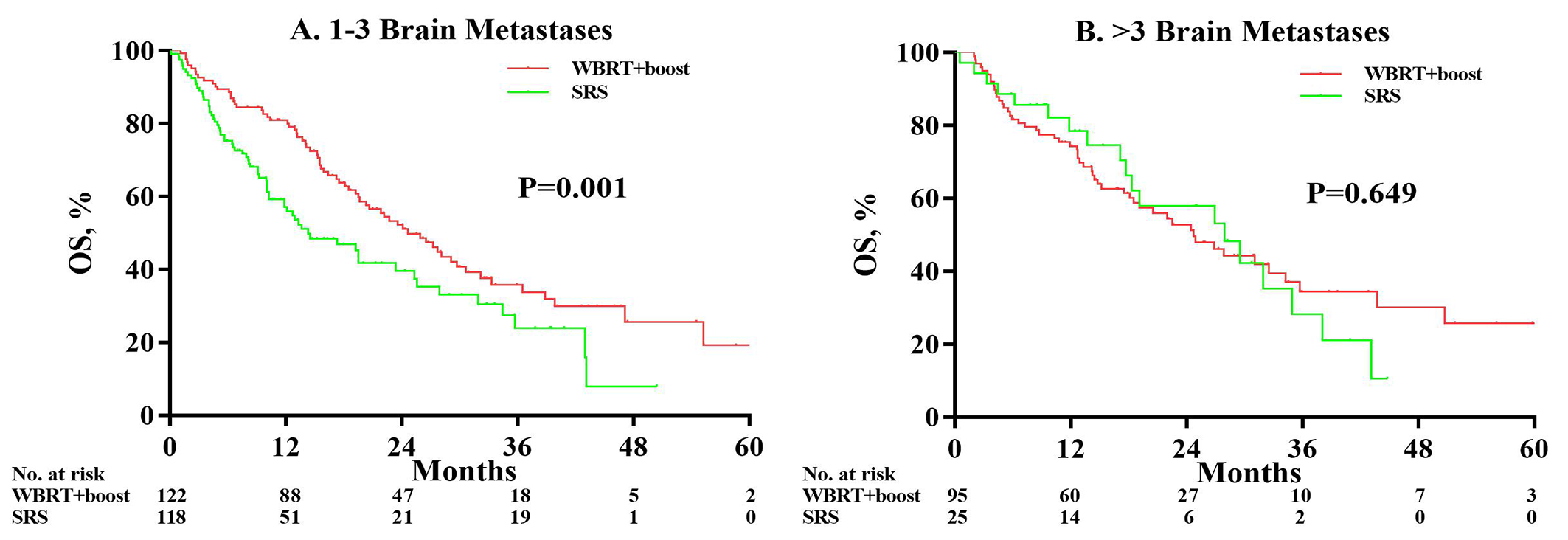

Supplement: Supplementary Figure 1 — Overall survival impact of number of brain metastases. Abbreviations: WBRT+boost indicates whole brain radiotherapy plus focal radiation boost; SRS, stereotactic radiosurgery. The plots showed that compared to SRS alone, a significant survival benefit of WBRT+boost in patients with 1 to 3 brain metastases (A) (24.6 vs 14.3 months, P = 0.001). [file Image_1.tif]

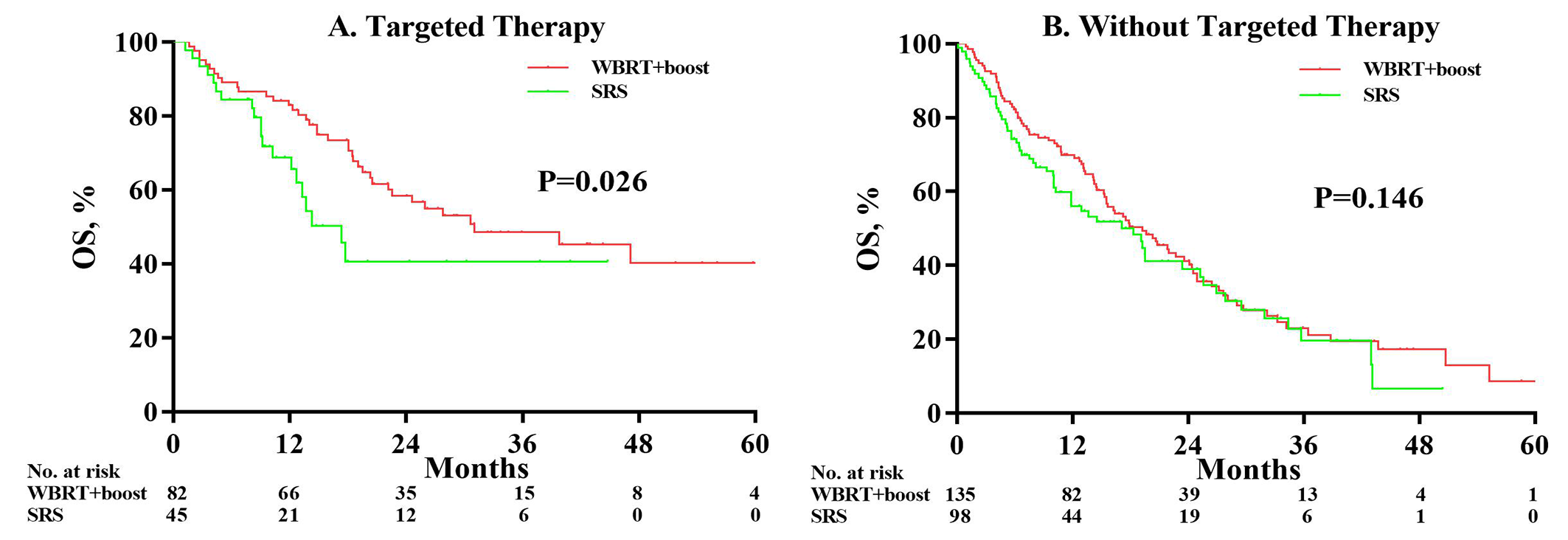

Supplement: Supplementary Figure 2 — Comparison of overall survival among subgroups based on the administration of systemic treatment. Abbreviations: WBRT+boost indicates whole brain radiotherapy plus focal radiation boost; SRS, stereotactic radiosurgery. The plots showed a significant difference in OS was observed in patients who received targeted therapy (A) in the comparison of groups (31.0 vs 17.3 months, P = 0.026). [file Image_2.tif]
